# Atomic Analysis of Protein-Protein Interfaces with Known Inhibitors: The 2P2I Database

**DOI:** 10.1371/journal.pone.0009598

**Published:** 2010-03-09

**Authors:** Raphaël Bourgeas, Marie-Jeanne Basse, Xavier Morelli, Philippe Roche

**Affiliations:** Laboratoire Interactions et Modulateurs de Réponses (UPR3243), Centre National de la Recherche Scientifique (CNRS) & Aix-Marseille Universités, Institut de Microbiologie de la Méditerranée (IMM), Marseille, France; Leeds Institute of Molecular Medicine, United Kingdom

## Abstract

**Background:**

In the last decade, the inhibition of protein-protein interactions (PPIs) has emerged from both academic and private research as a new way to modulate the activity of proteins. Inhibitors of these original interactions are certainly the next generation of highly innovative drugs that will reach the market in the next decade. However, *in silico* design of such compounds still remains challenging.

**Methodology/Principal Findings:**

Here we describe this particular PPI chemical space through the presentation of 2P2I_DB_, a hand-curated database dedicated to the structure of PPIs with known inhibitors. We have analyzed protein/protein and protein/inhibitor interfaces in terms of geometrical parameters, atom and residue properties, buried accessible surface area and other biophysical parameters. The interfaces found in 2P2I_DB_ were then compared to those of representative datasets of heterodimeric complexes. We propose a new classification of PPIs with known inhibitors into two classes depending on the number of segments present at the interface and corresponding to either a single secondary structure element or to a more globular interacting domain. 2P2I_DB_ complexes share global shape properties with standard transient heterodimer complexes, but their accessible surface areas are significantly smaller. No major conformational changes are seen between the different states of the proteins. The interfaces are more hydrophobic than general PPI's interfaces, with less charged residues and more non-polar atoms. Finally, fifty percent of the complexes in the 2P2I_DB_ dataset possess more hydrogen bonds than typical protein-protein complexes. Potential areas of study for the future are proposed, which include a new classification system consisting of specific families and the identification of PPI targets with high druggability potential based on key descriptors of the interaction.

**Conclusions:**

2P2I database stores structural information about PPIs with known inhibitors and provides a useful tool for biologists to assess the potential druggability of their interfaces. The database can be accessed at http://2p2idb.cnrs-mrs.fr.

## Introduction

In the last decade, the inhibition of protein-protein interactions (PPIs) has emerged from both academic and private research as a new way to modulate the activity of proteins (for an in depth review see Roche and Morelli [1]). Based on this new focus, it is now more and more commonly accepted that protein-protein complexes are an important class of therapeutic targets [2]. PPIs can be involved in a network of complex interactions that play a central role in various cellular events. These interactions control processes involved in both normal and pathological pathways, which include signal transduction, cell adhesion, cellular proliferation, growth, differentiation, viral self-assembly, programmed cell death and cytoskeleton structure (for a review refer to [3]).

In parallel to this new field, large scale genomics and proteomics programs have permitted the identification of entire protein networks interactomes at the cellular level. These programs have led to major breakthroughs in understanding biological pathways, host-pathogen interactions and cancer development. With the growing tools of small molecules, the modulation of these networks of interactions represents a promising therapeutic strategy. Protein-protein interaction inhibitors (2P2Is) are certainly the next generation of highly innovative drugs that will reach the market in the next decade.

As a consequence of this enthusiasm, the exponential increase of published biomedical literature on PPIs and their inhibition has prompted the development of internet services and databases that help scientists to manage the available information. There is now a growing number of structural databases dedicated to protein-protein interactions [4]–[7]. A large variety of these PPIs databases depict protein-protein interactions at a structural level (for a summary of these available databases refer to [1]), but they focus only on this particular interface without taking into account the potential inhibitors related to one of the two partners. In a recent survey, Higuerueolo *et al.* analyzed the atomic interactions and profile of small molecules disrupting PPIs in the TIMBAL database, focusing on small molecules properties and comparing these results to drug-like databases [4]. Several other studies have also focused on subsets of small molecules that disrupt PPIs [5], [6], [7], [8]. However, none of them have focused on both the protein-protein structural information available and the known inhibitors within the interface.

We describe here a chemical space, 2P2I_DB_, which is a hand-curated database dedicated to the structure of Protein-Protein complexes with known inhibitors thereby offering complementary information to these previous analyses (2P2I_DB_ is available at http://2p2idb.cnrs-mrs.fr). We have analyzed the protein/protein and protein/inhibitor interfaces in terms of geometrical parameters, atom and residue properties, buried accessible surface area and other biophysical parameters, such as the protein-protein dissociation constant (Kd) of a complex. The interfaces found in 2P2I_DB_ were then compared to those of representative datasets of heterodimeric complexes from Bahadur and Zacharias [9] or from the ProtorP parameters (http://www.bioinformatics.sussex.ac.uk/protorp/ and [10].

The architecture present at the interface generally involves a globular interacting domain, a single secondary structure element (alpha-helix or beta strand) of a globular protein, or a short peptide. Complexes in 2P2I_DB_ present globally the same shape (planarity or eccentricity) than standard heterodimeric complexes, but their accessible surface areas are significantly smaller. More strikingly, no major conformational changes are observed between the different states of the proteins (bound to the biological partner, the equivalent free form and the form bound to the small molecule inhibitor). The interfaces are also more hydrophobic than general PPIs' interfaces, with less charged residues and more non-polar atoms. Moreover, fifty percent of the complexes in the 2P2I_DB_ dataset possess more hydrogen bonds than typical protein-protein complexes. A set of key descriptors were identified to distinguish between PPIs with known inhibitors and representative transient complexes in the protein databank. Transient protein-protein complexes are defined as protomers that, *in vivo*, can exist either on their own or in complex and also undergo an exchange between the free and complexed form [11].

A new classification based on these parameters is proposed with potential aims for the future to identify potential new druggable PPI targets.

## Results and Discussion

### Dataset Collection

**Figure 1:**
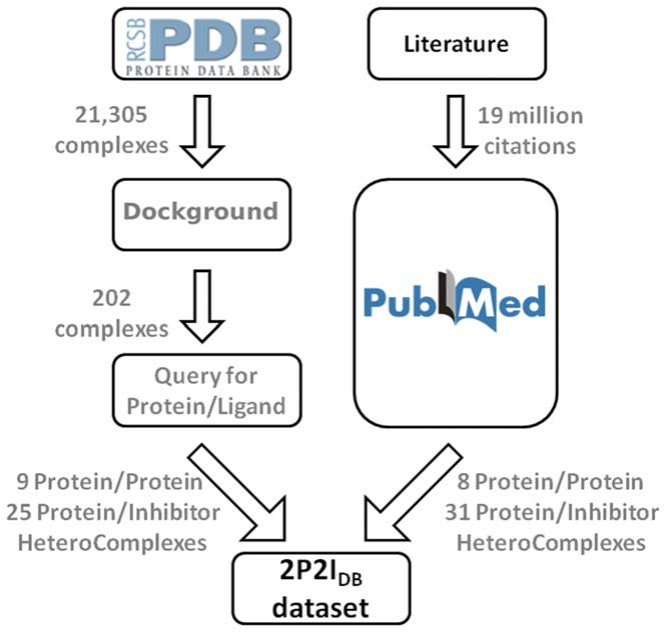
Flow chart indicating how the 2P2I_DB_ dataset was build from data mining. Two separate approaches were used to retrieve protein/protein complexes with known inhibitors for which structural information was available. The protein databank was search through the Dockground server [23] which led to 202 complexes that were filtered using an advanced query and manual inspection of the interface to give 9 protein/protein complexes and 25 protein/inhibitor complexes. Exhaustive search of the literature led to the discovery of 8 protein complexes corresponding to 31 protein/ligand complexes.

**Figure 2:**
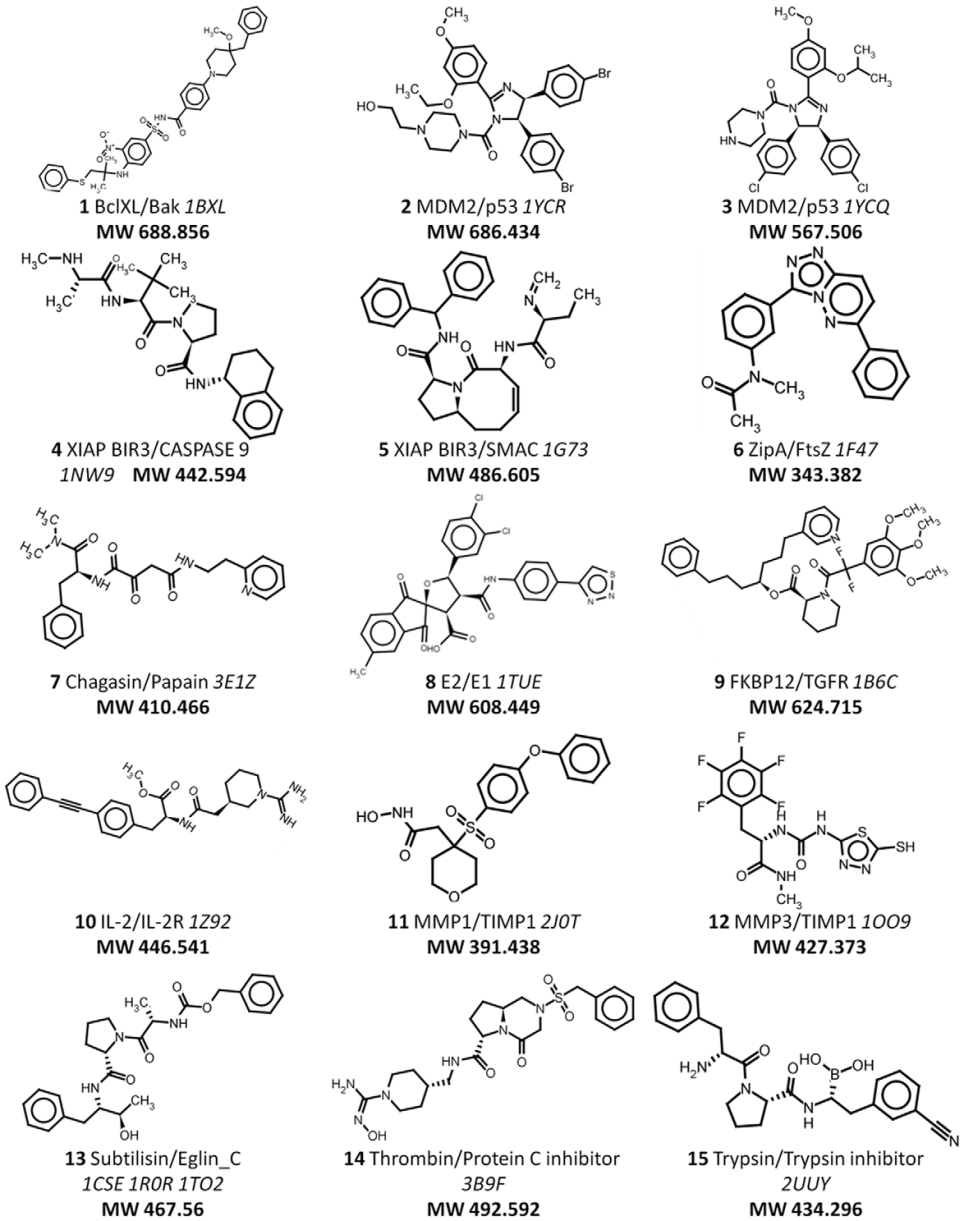
List of representative small molecule inhibitors for each protein/protein complex in 2P2I_DB_. Only the inhibitor used to define the subset of the interface at 4.5 Å around the ligand is shown. For each inhibitor, the name of the protein family, the PDB code of the complex and the molecular weight of the ligand are indicated.

As our goal was to define structural parameters that guide the development of PPI disruptors, we only considered those protein families for which a high resolution three dimensional structure was available for both the protein/protein and the protein/inhibitor complexes. Homodimers and covalently bound inhibitors were not taken into account due to their different behavior. When available, the best resolution structure of the unbound form of the proteins or a close homologue was included. The dataset was built through data mining from the literature and by exhaustive search of the Protein Data Bank (Figure 1 and Material & methods). The final dataset was compiled into a relational database (2P2I_DB_) that was used to further analyze the general properties of protein/protein interfaces (PPIs) with a known inhibitor. The 2P2I_DB_ (http://2p2idb.cnrs-mrs.fr) contains a total of 17 protein/protein complexes corresponding to 14 families and 56 small molecule inhibitors bound to the corresponding target (Table 1 and figure 2).

**Table 1:** Complex families in 2P2I_DB_.

Class	#	Family	Complex^a^	Number of Inhibitors^b^	Source^c^	Affinity^d^ (nM)	Ref
I	1	BclX_L_/Bak	1bxl	8	PubMed	340	[27]
I	2	MDM2/p53	1ycr 1ycq	3	PubMed	600	[28]
I	3	XIAP BIR3/CASPASE 9	1nw9	2	PubMed	20	[29]
I	4	XIAP BIR3/SMAC	1g73	5	PubMed	420	[29]
I	5	ZipA/FtsZ	1f47	4	PubMed	20,000	[30]
II	6	Chagasin/Papain	3e1z	1	PDB	0.036^e^	[31]
II	7	E2/E1	1tue	1	PubMed	na	[32]
II	8	FKBP12/TGFR	1b6c	17	PDB	na	[33]
II	9	IL-2/IL-2R	1z92	8	PubMed	10	[34]
II	10	MMP1/TIMP1	2j0t	1	PDB	0.40^e^	[35]
II	11	MMP3/TIMP1	1oo9	1	PDB	0.22^e^	[36]
II	12	Subtilisin/Eglin C	1cse 1r0r 1to2	1	PDB	0.029	[37]–[39]
II	13	Thrombin/Protein C inhibitor	3b9f	1	PDB	na	[40]
II	14	Trypsin/Trypsin inhibitor	2uuy	3	PDB	0.02	[41]

PPIs were subdivided into class I that correspond to protein/peptide interactions with less than six segments at the interface (families 1–5) and class II that represent more globular interacting domains with more segments (families 6–14).

a PDB code of protein/protein complexes.

b Number of inhibitors present in the database for a given protein/protein complex.

c Structures were retrieved through exhaustive search of the protein databank (PDB) or literature data mining (PubMed).

d Dissociation constant (K_D_) of the protein/protein complexes are indicated in nanomolar when available.

e indicates Ki values.

There are a limited number of targets in the 2P2I database at this stage due to the structural prerequisites that were used. However, it is inevitable that high throughput structural genomic programs will generate a high level of data. In addition, the development of improved methodologies for the development of small molecule inhibitors will rapidly lead to the discovery and structural characterization of disruptors of new PPI families. These new targets and their corresponding ligands will be incorporated into the database as they appear in the literature and the Protein Data Bank (http://www.rcsb.org/).

To assess the characteristics of druggable PPIs, the general properties of the interfaces found in 2P2I_DB_ were compared to those of representative datasets of heterodimeric complexes retrieved from Bahadur and Zacharias [9] and from the ProtorP server [10].

### Global and Local Rearrangements

#### PPI with known inhibitors do not undergo large conformational changes

The formation of heterodimeric complexes can lead to large rearrangements of the two protein partners [12]. To assess this point, we measured the root mean square deviation (rmsd) between the bound partners, the equivalent free forms and the form bound to a small molecule inhibitor for each complex family (Table 2 and Table S1). Strikingly, complexes stored in 2P2I_DB_ only underwent minor local adaptation during complex formation. The average rmsd (1.12±0.4 Å) was not significantly different than the natural conformational dynamics of the free target protein and was in the same range as the resolution of the crystal structures. Some local rearrangements could be observed at the binding site; however, these rearrangements do not impair the possibility to design potent inhibitors with high affinity. The fact that there is no main rearrangement between the different forms in the 2P2I_DB_ dataset could mean that these types of complexes are easier to target. Other strategies with small molecule inhibitors binding at different sites, such as an allosteric pocket away from the interface, would be necessary to disrupt PPIs with large conformational rearrangements. These classes of inhibitors are not present in the 2P2I_DB_, as we only kept those ligands that are present at the interface.

**Table 2:** General interface parameters of PPIs in the 2P2I_DB_ dataset.

	PDB	Plan (Å)^a^	Ecc^b^	SecS^c^	GV (Å^3^)^d^	GV_I (Å)^e^	Hb^f^	SB^g^	ASA (Å^2^)^h^	H_ASA (%)^i^	RMSD (Å)^j^	Pockets (Å^3^)^l^	Seg^m^
**BclXL/Bak**	1bxl	3,60	0,75	H/H	2892	1,75	0,05	0	825	45,6	2,3	97	5
**MDM2/p53**	1ycr	2,99	0,86	H/H	786	0,60	0,34	1	660	54,2	1,9	351	3
**MDM2/p53**	1ycq	2,14	0,62	H/H	1286	1,38	0,39	1	455	65,7	1,8	215	2
**XIAP BIR3/CASPASE 9**	1nw9	2,32	0,78	S/S	3567	1,79	0,78	0	241	100,0	2,4	244	2
**XIAP BIR3/SMAC**	1g73	2,18	0,73	S/S	3500	5,35	0,88	0	472	88,9	2,3	140	3
**ZipA/FtsZ**	1f47	2,76	0,75	S/H	3503	3,24	0,09	0	541	39,2	0,9	0	5
***mean***		***2,66***	***0,75***	***-***	***2589***	***2,35***	***0,42***	***0,33***	***532***	***65,6***	***1,9***	***174,5***	***3.3***
***standard dev.***		***0,57***	***0,08***	***-***	***1238***	***1,70***	***0,34***	***0,52***	***198***	***24,3***	***0,6***	***122,7***	***1.4***
**Chagasin/papain**	3e1z	3,00	0,89	C/C	4286	2,26	0,53	0	947	55,4	0,4	279	9
**E2/E1**	1tue	2,59	0,71	H/H	5042	2,86	0,55	3	946	32,6	1,4	202	7
**FKBP12/TGFR**	1b6c	2,82	0,42	S/H	5457	3,14	0,17	0	869	57,2	0,5	387	8
**IL-2/IL-2R**	1z92	2,40	0,86	H/C	4431	2,47	0,7	5	898	39,8	1,3	146	8
**MMP1/TIMP1**	2j0t	2,76	0,55	C/C	5380	4,08	0,83	0	660	48,8	0,5^k^	323	7
**MMP3/TIMP1**	1oo9	3,02	0,78	C/C	5157	2,76	0,24	0	936	39,7	1,2^k^	227	9
**Subtilisin/Eglin C**	1cse	2,67	0,65	C/S	3858	3,01	0,74	0	640	62,6	0,3	282	8
**Subtilisin/Eglin C**	1r0r	2,54	0,75	C/C	3763	2,99	0,78	0	630	66,3	0,3	230	9
**Subtilisin/Eglin C**	1to2	3,11	0,61	C/S	3277	2,25	0,74	1	728	66,4	0,3	275	8
**Thrombin/Protein C inhibitor**	3b9f	3,41	0,81	C/C	5538	4,33	0,72	2	639	38,0	0,6	350	9
**Trypsin/trypsin inhibitor**	2uuy	2,57	0,73	C/C	3500	3,12	1,09	1	562	70,1	0,7	154	10
***mean***		***2,81***	***0,71***	***-***	***4517***	***3,02***	***0,64***	***1,09***	***769***	***52,4***	***0,7***	***259,6***	***8.4***
***standard dev.***		***0.30***	***0.14***	***-***	***836***	***0,67***	***0,26***	***1,64***	***151***	***13,3***	***0,4***	***76,7***	***0.9***

a Planarity;

b eccentricity;

c secondary structure elements at the interface for the target and related partner;

d Gap volume;

e Gap volume index;

f Hydrogen bonds per 100 Å^2^ of interface;

g Salt bridges;

h Accessible surface area buried at the interface of the protein/protein complex;

i Accessible surface area hidden by the inhibitor;

j Root mean square deviation (CA atoms) between unbound protein and complex;

k when unbound protein was not available, rmsd between protein/protein and protein/ligand complexes was computed;

l Total pocket volume at the interface;

m Number of interface residue segments. For each parameter the mean and standard deviation are presented.

### General Properties of the Interacting Partners

#### PPI with known inhibitors can be divided into two classes

We analyzed the general characteristics of the protein/protein interfaces (PPIs) using online servers [10] or local visualization programs. The 17 complexes could be divided into two classes according to the number of continuous segments at the interface (Tables 1 & 2). Class I contained six of the 17 PPIs (Table 1, families 1 to 5) and used a limited number of continuous segments for binding to the partner (average value 3.3±1.4). Interestingly, small peptides are able to mimic the interacting partner for this class of complexes (Table 1, families 1 to 5 and references therein). The remaining eleven PPIs used a high number (average value of 8.4±0.9) and corresponded to actual globular interacting domains (complex families 6–13 in Table 1).

A more detailed analysis revealed that complexes from class I contained a higher proportion of secondary structure elements at the interface. Four out of 6 complexes involved mainly an alpha helix at the interface, and the other two a beta strand (see supplementary material, Table S2). Class I PPIs involved a well ordered partner, which might be easier to mimic with small molecules. This later observation could account for the greater number of inhibitors developed for this type of interface [4]. Alternatively, PPIs from class II contained a higher proportion of nonstructured elements probably due to their larger size. However, it is noteworthy that the difference in nature of these two sets of PPIs does not seem to affect the size of the small molecule inhibitors because similar molecular weight ranges and averages were observed in our dataset for the two classes (data not shown).

When available, dissociation (K_D_) or inhibitory (K_i_) constants of the protein/protein complexes were compared (Table 1). On average, class I complexes corresponded to low affinity complexes in the micromolar range, whereas class II complexes revealed a higher affinity in the nano or sub-nanomolar range.

Subsequent analyses were performed on the two classes of complexes including analysis of the protein/protein and protein/inhibitor interfaces in terms of geometrical parameters, atom and residue properties, and buried surface area at the interface.

### Geometry of the Interfaces

#### PPI with known inhibitors are smaller than standard heterodimers

The size of the interface was computed for each PPI by measuring the buried surface area between the protein/protein complexes and the unbound proteins. The average interface area of 685.2±200 Å^2^ (ranging linearly from 241 to 947 Å^2^) was significantly smaller than the standard average values of approximately 1000 Å^2^ observed for heterodimeric protein-protein complexes, as described in the literature by Bahadur and Zacharias [9] and on the ProtorP server [10]. The average values of 532±198 Å^2^ and 769±150 Å^2^ were observed for class I (families 1–5 in Table 1) and class II (family 6–13 in Table 1) complexes respectively. The average area of the interface is smaller when the interaction involves a short peptide segment. Moreover, this analysis also illustrate that interfaces with a known PPI inhibitor are slightly smaller than overall protein-protein complexes. However, the 17 interfaces analyzed cover a wide linear range in terms of size, which indicates that the size of the interface does not thwart the definition of a potential target.

#### PPI with known inhibitors share geometrical properties with heterodimers

The average planarity of interfaces in our dataset was 2.8±0.4 Å, which is a value equivalent to that of overall heterodimeric complexes (2.7±1.2 Å). Similarly, eccentricity (*i.e.,* the ratio of the lengths of the principal axes of the least-squares plane through the atoms in the interface) was not significantly distinguishable (0.72±0.12 vs. 0.70±0.12). Similar values were observed for the two classes defined above. The Gap volume index (GVi) provides a measure of the tightness of a protein-protein complex [13]. The average GVi values for our dataset and general protein-protein complexes are 2.8±1.1 and 2.8±1.4, respectively. On average, a tighter fit was observed for class I complexes (2.3±1.7), which could be due to their smaller size. However, a large variability was observed between complexes. The surface complementarity between the two partners was not correlated with the binding affinity, which could be accounted for by the entopic and desolvation terms of the binding energy. The chemical nature of the interface plays a more important role in defining the strength of the interaction.

Overall, these results strongly suggest that PPIs with known inhibitors share similar shape properties than average transient protein-protein complexes.

#### PPIs with known inhibitors posses few pockets at the interface

PPIs and PPI inhibitor interactions use a greater number of small pockets than protein-ligand interactions [14]. The number and size of pockets at the interface for all the targets in 2P2I_DB_ were calculated using Q-SiteFinder [15]. Average values of 1.8±0.7, 1.6±0.7 and 1.7±0.6 pockets corresponding to active volumes of 360±244 Å^3^, 303±160 Å^3^ and 247±86 Å^3^ were found for the target proteins in their free form, bound to the inhibitor and bound to their interacting partner, respectively. Similar values were observed for the two classes of PPIs. In their original paper, Fuller *et al.* reported 6±3 pockets for PPIs with an average size of 54 Å^3^. However, they calculated 99 pockets for each protein surface and only 10 could be visualized on the Q-SiteFinder web server. Because only the larger pockets could be visualized in that study, this limitation could account for the slightly smaller number of pockets observed in our dataset compared to those reported in the literature [14]. ZipA was not included in the general statistics because modulators of ZipA/FtsZ possess a remarkable way of binding and they do not penetrate the ZipA surface (supplementary material, Fig. S1). As a consequence, no pocket was found at the interface for this target.

We then analyzed the protein/protein and protein/inhibitor interfaces in terms of chemical properties.

### Chemical Nature of the Interface

#### More hydrogen bonds at the interface

Hydrogen bonds play a key role in the specificity of the interaction between two proteins. The number of hydrogen bonds per 100 Å^2^ of interface was estimated for the different complexes of the 2P2I_DB_ dataset. The average number of hydrogen bonds was comparable to that reported for protein-protein complexes (0.56 vs 0.52, [9]). On average, PPIs involving a peptide at the interface (class I) possessed less hydrogen bonds (0.42) than globular PPIs (class II, 0.64). However, large variations were observed for individual complexes; the average number of hydrogen bonds per 100 Å^2^ varied from 0.05 to 1.1. Fifty percent of the complexes in the 2P2I_DB_ dataset possessed between 0.69 and 0.83 hydrogen bonds per 100 Å^2^ of interface indicating that the majority of 2P2Is possess more hydrogen bonds than typical protein-protein transient complexes (supplementary material, Fig. S2). Moreover, we analyzed the portion of the protein-protein interface that is occupied by the inhibitor in the protein/ligand form and found that for the majority of the PPIs, a large number of hydrogen bonds where located in that region.

#### Less salt bridges at the interface

PPIs with known inhibitors possess between zero and two salt-bridges, which are directly located in the region that is disrupted by the ligand (Table 2). The equivalent number for transient heterodimers could not be extracted from the literature, but it is very likely to be higher than the observed value in our dataset. The interaction between IL-2 and its receptor is an exception, as it contains five salt bridges with two of them corresponding to the binding site of the inhibitor. Interestingly, among the eight known inhibitors, six possess a guanidinium group that mimics a key arginine (Arg36) of the partner involved in one of the two salt bridges with a glutamic acid (Glu62) of the target.

#### Less charged residues at the interface

On average, PPIs with known inhibitors contain less charged residues at the interface (18.9±13.8%) than standard transient heterodimers (27.0±12.5%). This result indicates that PPIs with known inhibitors are more hydrophobic than typical heterodimers, which can be correlated to the observation that small molecule PPI modulators are hydrophobic [4]. However, a wide range of situations was observed with percentage values (from 0 to 46 percent), which confirms that PPIs with known inhibitors cover a diverse area of chemical space.

### Descriptors of the Interface

**Figure 3:**
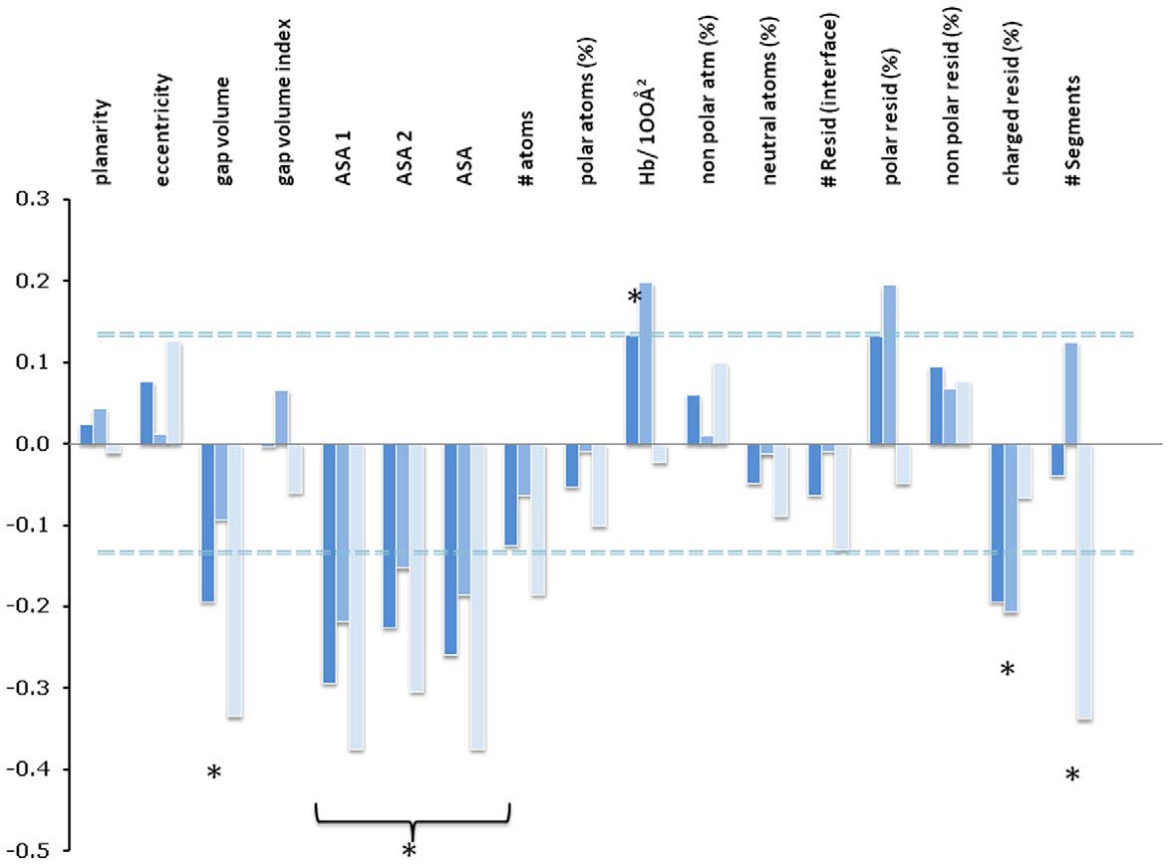
Student t-test allowing the selection of three main discriminating parameters for the PCA analysis. The t-test was calculated for the whole dataset and for each class (I and II) separately. Dotted lines indicate a threshold of confidence higher than 90% to differentiate 2P2I_DB_ complexes from transient heterodimers. On the basis of this analysis, ASA, Gap volume, Number of interfacial segments, hydrogen bonds and the percentage of charged residues were selected for the PCA analysis.

We used a student's t-test to compare the main descriptors of PPIs in our dataset to standard heterodimers in an attempt to extract the most discriminating parameters that govern the druggability of a PPI. The number of interfacial segments, accessible surface area (ASA), Gap volume, hydrogen bonds and the percentage of charged residues were selected as the key parameters that typify PPIs with known inhibitors (Figure 3). The difference between the 2P2I_DB_ dataset and transient heterodimers for the four selected parameters corresponded to probabilities higher than 90% confidence according to the *t-distribution* table of significance.

#### PCA analysis

**Figure 4:**
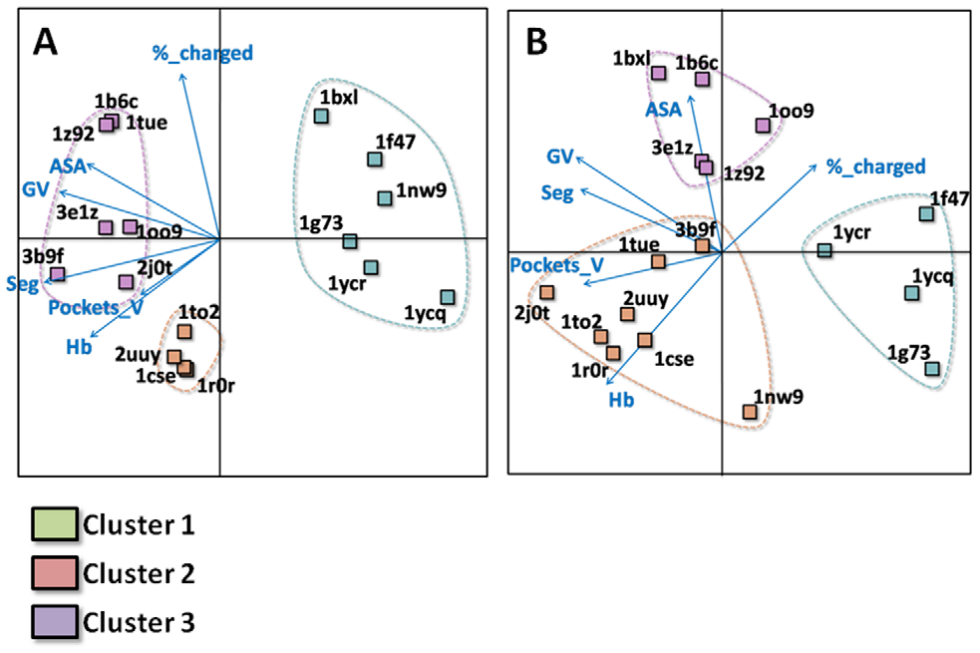
PCA analysis. Six key parameters were selected to perform the PCA analysis to separate the complexes into different groups: The five parameters based on the t-test and defined in figure 3 as well as total pocket volume at the interface. **A:** Analysis on the whole interface. Three different PPI clusters were defined. Cluster 1 (green) regrouped all complexes from class I corresponding to targets interacting with a peptide. Subtilisin and trypsin complexes defined cluster 2 (pink). Cluster 3 (purple) regrouped all other protein/protein complexes. **B:** Same analysis done on the part of the interface that is 4.5 Å around the ligand. The protein/protein complexes were in three slightly different clusters. Four out of six class I complexes were grouped together. Subtilisin and trypsin complexes remained very closely associated.

In addition to the five parameters defined above (ASA, Gap volume, number of segments at the interface, hydrogen bonds and percentage of charged residues), the combined pocket volumes at the interface were used in a principal component analysis (PCA) to separate the 2P2I_DB_ dataset into different families. Pockets volumes were not incorporated in the t-test study because statistics were not available to compare to the transient protein-protein dataset. However, they were used in the PCA analysis because they are known to play an important role in protein-protein specific interaction [14]. Out of the six parameters, four corresponded to geometric descriptors of the interface (ASA, Gap volume, number of interfacial segments and pocket volume) and two to the chemical nature of the interface (percentage of charged residues and hydrogen bonds). The influence of each descriptor is indicated by the direction and length of the corresponding arrows (Figure 4). All selected parameters have an important contribution to the clustering procedure (length of arrows) and they cover a large analytical space (direction of arrows). Three groups were found as a result of the clustering (Figure 4A). Interestingly, all six complexes that belong to class I were grouped together (cluster 1, green), which validates our choice of parameters for the PCA analysis. Subtilisin/Eglin C and trypsin/trypsin inhibitor formed a second group, mainly based on ASA and charged residue parameters (cluster 2, pink). The remaining seven complexes were grouped in a third cluster (cluster 3, purple). Parameters characteristic of weak and strong transient dimers as defined by Nooren and Thornton [11] were incorporated in a new PCA analysis. The parameters representative of the weak and strong dimers were grouped with cluster 1 and 3, respectively (data not shown). Therefore the PCA analysis with the six selected key parameters of the interface can be used to predict the type of interaction of the PPI, which can be of particular interest to define future targets for the discovery of new PPI inhibitors.

A similar PCA analysis was performed with parameters derived from the subset of the interface that is in direct contact with the inhibitor in the protein/ligand structure (Figure 4B). The same descriptors were used as mentioned above. Again, three clusters were identified; however, the separation between class I and class II was not as clear as for the whole interface. Two out of six complexes from class I behaved differently. In the case of the XIAP/Caspase complex (PDB code: 1nw9) the high number of hydrogen bonds and the absence of charged residues were responsible for its different behavior. In the BclX_L_/Bak complex (PDB code: 1bxl), the high values for ASA and the number of segments and low hydrogen bond content led to its classification next to the FKBP12/TGFR complex (PDB code: 1b6c). Subtilisin/Eglin C and trypsin/trypsin, which formed a separated cluster when considering the whole interface, were grouped together when considering the part of the interface that interacts with the ligand.

### 2P2I Web Server Description

**Figure 5:**
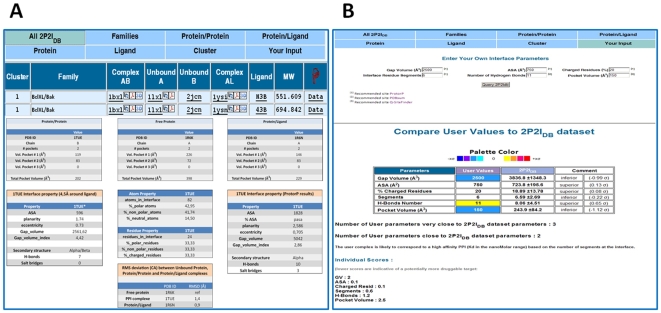
2P2I_DB_ Server (http://2p2idb.cnrs-mrs.fr). **A:** Query procedures to retrieve data stored in the database for each PPI. The database can be searched by family, by PDB codes of the protein/protein complexes, protein/ligand complexes, or unbound proteins, ligand properties or cluster number. A list of PDB codes or Uniprot numbers is returned as well as links to relevant databases. A detailed report of properties of each PPI is also provided. **B:** Interactive menu to compare user defined PPI properties with the 2P2I_DB_ dataset. The user can enter pre-calculated values for the six key descriptors that were used in the PCA analyses. In return, these parameters are compared to the 2P2I_DB_ dataset and an estimation of the druggability of the target is proposed.

A web server was developed to facilitate the access to the data calculated for the different PPIs. It allows the user to search for specific complexes using different query procedures based on families, pdb codes, Uniprot numbers, cluster family or ligand properties (Figure 5A). For each query, a table is returned giving a list of PPIs matching the query. For each protein/protein complex, the cluster number, the family name, the pdb codes of the protein/protein complex, the unbound partners and the protein/inhibitor complex, the three letter code of the ligand and its molecular weight are given. Links to relevant databases and to literature are also provided. Finally, a data report containing the main analyses of PPIs can be accessed in different tabs.

An interactive menu allows the user to compare the properties of a given PPI to the 2P2I_DB_ dataset using the six key descriptors that were selected for the PCA analyses (Figure 5B). The parameters should be pre-calculated using online web servers (ProtorP [10], PDBSum [16] or Q-SiteFinder [15]) and entered on the 2P2I_DB_ website. Each parameter is compared to the equivalent parameter in the 2P2I_DB_ dataset and the number of parameters that are close or very close to 2P2I_DB_ parameters is returned. An indication of the type of PPI in terms of binding affinity (weak or strong) is also provided. Finally, individual scores for each parameter are calculated. Lower values are indicative of complexes with high potential to become druggable targets whereas higher scores are likely to correspond to poorly druggable complexes.

### Conclusions

PPIs interfaces have long been considered to be poorly druggable because of their general properties. However, in the last few years, a growing number of PPI inhibitors have been discovered, and some of these inhibitors have even reached the preclinical stage of investigation [8], [17]. The number of currently available drug targets is very limited [18]. Due to their crucial role in various biological processes and in the dysfunction of cells, PPIs will probably become the next generation of successful therapeutics.

To this critical question *“What makes a PPI an attractive target for the discovery and development of small molecules?”* we can portray the good PPI candidates in a few points, which are summarized from existing published data, as follows: i) The target should be validated at least biologically (RNAi, knock-out gene etc…) or, even more importantly, clinically, and the inhibition of the PPI should not be associated with toxicity; ii) Key residues involved in the interaction and defining hot spots should be characterized through mutagenesis analyses, alanine scanning or by server prediction; iii) Structural information on the PPI complex and/or unbound forms should be available to accelerate the process; bridging water molecules should be taken into account when available and because the best conformation to target for PPI inhibition is not necessarily the one found in the complex, the natural conformational dynamics of the target should be investigated through molecular dynamic simulations [19], normal mode analysis [20], conformation ensemble predictions [21] or NMR [22]; iv) Three to five pockets should be available at the interface in the free form or in the simulated conformations of the target for a total volume of at least 250 Å^3^ [14].

A recent study concluded that small molecule PPI modulators are larger (average molecular weight >400 Da), more hydrophobic (average alogP ∼4), with more aromatic rings (∼4 in average) and make fewer hydrogen bonds with the protein than average drugs [4]. However, a detailed description of the chemical and topological spaces of protein interfaces that can be disrupted by small drugs was not available. The present study leads to a better definition of a potentially successful PPI target.

We have gathered information available for 17 PPIs with known inhibitors whose three dimensional structural had been characterized. The interfaces were analyzed in terms of geometrical parameters, shape and chemical properties. The protein/protein complexes could be divided into two classes according to different parameters, such as the number of segments at the interface. Class I PPIs correspond to those that interact with peptide-like partners and show more secondary structure elements at the interface, whereas the class II group comprises more globular protein/protein complexes with more unstructured elements at the interface.

The different PPIs were further classified by PCA analysis using descriptors that were selected based on t-test evaluations and general analyses of the interfaces. Six interfacial parameters were selected corresponding to ASA, Gap volume, percentage of charged residues, number of segments, hydrogen bonds and total volume pockets. Three clusters were defined as a result of the PCA analysis; cluster 1 corresponded to class I PPIs, while class II PPIs were subdivided into two clusters.

Analysis of the part of the PPI that corresponds to the region directly in contact with the inhibitor led to similar results. However, minor differences could be observed, which suggests that parameters that define the druggability of a target are probably different from the parameters that define the chemical space of PPI inhibitors. The descriptors were selected for their ability to discriminate between whole PPIs with known inhibitors and transient dimers; additional parameters would have to be selected to predict the chemical space of the ligands that are likely to disrupt a given PPI.

The number of structurally characterized complexes with known inhibitors is small. Therefore, the chemical space of PPIs is not completely covered in the 2P2I_DB_ because of the limited amount of data currently available. However, the definition of what makes a PPI a potentially druggable target will become more and more reliable as the number of 3D structures increases.

The ZipA example, in which the ligand is lying on the protein surface in an unconventional way (supplementary material, Fig. S1), highlights the difficulty of defining general parameters for the druggability of PPI targets. However, we are at an early stage in the process of defining new relevant PPI targets and, as for HTS approaches, the goal is to be able to improve the selection of new PPI targets rather than define all the potential targets.

Further improvements will include incorporating other parameters such as available mutation data, known or predicted hot spots, dynamic behavior of the interface and the development of new databases dedicated to other types of PPI (such as disordered interacting segments and PPIs with large domain rearrangements, which will need specific descriptors).

The results of our study serve to expand current knowledge with new data and focuses at the interface of protein/protein complexes with prior structural knowledge. The proposed classification should lead to a better definition of potentially successful PPI targets and will accelerate the process of designing new PPI drugs. As successes in discovering PPI inhibitors accumulate, the parameters will be refined and the classification scheme updated.

The whole 2P2I dataset was organized as a relational database and can be accessed through a publicly available web server (http://2p2idb.cnrs-mrs.fr).

## Materials and Methods

### Dataset Collection

The hand curated dataset was constructed using two parallel approaches:

#### Search from the PDB

The entire PDB was searched using Dockground server [23] in order to collect protein-protein complexes. Crystal structures of heteromultimeric complexes with a resolution of 2.0 Å or lower were retrieved. Disordered protein and complexes with nucleic acid were discarded. A total of 202 heteromultimeric complexes were obtained.

The whole Protein Data Bank was then searched using an advanced query for free protein structures corresponding to each complex bound to small molecule inhibitors (supplementary material, Fig. S3). We then manually checked that the inhibitor was present at the interface between the two proteins and not covalently bounded to the protein. Nine protein/protein and 25 protein/ligand complexes were finally retrieved (Table 1, Source = PDB). When available, the unbound proteins were also included in the database.

#### Literature data mining

Eight protein/protein and 31 protein/ligand were retrieved by an exhaustive search of the literature (Table 1, Source = PubMed). IL-2/IL-2R and MDM2/p53 families were retrieved directly from the work of Pagliaro *et al.* [7]. Other complexes (BclX_L_/Bak, E2/E1, XIAP BIR3/Caspase, XIAP BIR3/SMAC, ZipA/FtsZ) were not found in the PDB search either because the interacting partner was a peptide or because of the x-ray resolution (>2 Å).

The two lists were combined to form the final dataset, which can be downloaded at http://2p2idb.cnrs-mrs.fr/dataset/2P2Idataset.zip. The whole dataset is composed of 17 protein-protein complexes, 23 unbound proteins and 64 protein-inhibitor complexes. The PDB IDs are: 1bxl, 1lxl, 1ysi, 1ysn, 2o2m, 1ysg, 2o2n, 2o22 (BclXL/Bak); 1z1m, 1ttv (MDM2/p53); 1tfq, 1tft (XIAP_BIR3/CASPASE_9); 1f9x (XIAP_BIR3/SMAC); 1oo9 (MMP3/TIMP1) corresponds to solution NMR structures. All other PDB codes correspond to x-ray structures.

### 2P2I Database

The protein-protein interaction inhibition relational database was developed with MySqL. It stores information about the 17 PPIs described in this study. Scripts for interaction with the DB have been developed in PhP with the software MyAdmin.

### Web Server

The 2P2I_DB_ database can be accessed through a web-based user interface (http://2p2idb.cnrs-mrs.fr). This platform allows users to query the database to get structural information about interfaces of stored complexes. The whole database can be searched by protein family, PDB codes of the free proteins, protein-protein or protein-ligand complexes, UniProt numbers, ligand three letter codes or by cluster number.

The user can also provide key parameters calculated from other web resources to compare the property of a given PPI to the 2P2I dataset.

### Dataset Analysis

#### Root mean square deviations

The root mean square deviations (rmsd) between free and bound states of different proteins were computed over CA atoms using DaliLite server (http://www.ebi.ac.uk/Tools/dalilite/index.html) and are summarized into Table S1.

#### Geometrical parameters

Planarity, eccentricity, secondary structure in interface, Gap volume, Gap volume index, number of atoms in interface, % polar atoms in interface, % non polar atoms in interface, % neutral atoms in interface, number of residues in interface, % polar residues in interface and % non polar residues at the interfaces were calculated with the ProtorP server using default parameters (http://www.bioinformatics.sussex.ac.uk/protorp/). Continuous interface segment have been defined in the literature as a stretch of residues that starts and ends with interface residues and may contain intervening non-interface residues. While considering the length of the segment, only interface residues are counted [13], [24].

#### Size of the interface

Accessible surface area (ASA) and percentage of charged residues were computed with the Naccess program with a probe radius of 1.4 Å. The size of the interface corresponded to the difference in ASA between the protein without its partner and in the complex.

#### Pocket size and volumes

Pockets at the interface were computed with the Q-SiteFinder server (http://www.bioinformatics.leeds.ac.uk/qsitefinder) on the protein-protein, protein-inhibitor complexes and the equivalent free proteins [14]. The homologous proteins were superimposed and only the pockets that were at least partly occupied by the inhibitor were retained.

#### Secondary structures

The percentage of secondary structure elements in the interface of the target protein and its partner were calculated with VMD using *in house* scripts. Four categories were defined for the overall class of the interface: H: Alpha Helix >30% and Beta strands <30%; S: Alpha Helix <30% and Beta strands >30%; HS: Alpha Helix >30% and Beta strands >30%; and Coil: Alpha Helix <30% and Beta strands <30%;

#### Hydrogen bonds and salt bridges at the interface

Hydrogen bonds were computed with the Pymol software (http://pymol.sourceforge.net/) using a 3.2 Å distance cutoff between the hydrogen atom and the acceptor atom and were checked manually.

A salt bridge was considered when an acidic residue (Asp or Glu) on one side of the interface and a basic residue (Arg, His or Lys) on the other side were less than 4.0 Å apart. Each putative salt bridge was then validated manually using VMD (http://www.ks.uiuc.edu/Research/vmd/).

#### Analysis of a subset of the interface

A subset of the interface was defined by taking into account only atoms around 4.5 Å of the ligand in the protein-inhibitor complexes (see supplementary material Table S3).

### Statistical Analyses and Clustering

#### T-test

The t-values were calculated as follows:
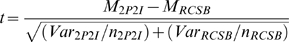



Where Var_2P2I_ and Var_RCSB_ are the variance of parameters in each group; M_2P2I_ and M_RCSB_ are means of these groups. The n_2P2I_ and n_RCSB_ are the total number of complexes in each group. Based on student's *t-distribution* table of significance (http://www.math.unb.ca/~knight/utility/t-table.htm), values higher than 1.34 correspond to probabilities of more than 90% confidence. On the one hand, if t-value is positive and greater than 1.34, then the mean of the studied parameter is significantly greater in the 2P2IDB dataset than in the RCSB transient dimers dataset at 90% or higher confidence level. On the other hand, if the t-value is negative and less than −1.34, then the mean of the studied parameter is significantly less in the 2P2I_DB_ dataset than in the RCSB transient dimers dataset.

#### PCA

Six parameters (ASA, Number of segments at the interface, Gap volume, pocket volume, hydrogen-bonds and the percent of charged residues in interface) were selected for the multivariate analysis performed according to the principal component analysis. Data were analyzed with the R software (http://www.R-project.org) and the ade4 package [25].

#### Clustering

Clustering of the PPIs into three groups was performed using the K-mean method [26].

## Supporting Information

Figure S1 ZipA protein in complex with IQZ inhibitor (PDB code 1S1J). The IQZ inhibitor ZipA surface is shown as a stick representation. (Figure generated with pymol).
(0.11 MB PDF)

Figure S2 Hydrogen bonds per 100 Å^2^ of accessible surface area. The X-axis represents the number of hydrogen bonds per 100 Å^2^. The Y-axis illustrates the number of complexes present in 2P2IDB having this number of hydrogen bonds (within ±0.1), i.e. y value at x = 0.6 indicates that there are 3 complexes having 0.3 to 0.5 hydrogen bonds per 100 Å^2^.
(0.09 MB PDF)

Figure S3 Advanced query search of the protein databank. This table lists the different parameters used to parse the protein databank to search for proteins bound to a small molecule inhibitor.
(0.08 MB PDF)

Table S1 Root mean square deviations for the complexes in 2P2I database. The rms are computed between the unbound protein and its equivalent in the protein/protein complex; the unbound protein and its homologous in complex with the inhibitor; the protein in complex with its partner and the homologous in complex with the inhibitor. When several structures are compared, the average is shown. All RMSD were performed over CA atoms with the DaliLite web server.
(0.15 MB PDF)

Table S2 Secondary structure at interface. This table lists secondary structures at interface (as defined in M&M) for each complex present in 2P2IDB. Information is detailed for both the target protein and its partner.
(0.16 MB PDF)

Table S3 Geometrical and chemical parameters for the subset of the interface at 4.5 Å around the inhibitor. The parameters are detailed for each complex of 2P2IDB and mean and standard deviations are shown for class I, class II and the whole database.
(0.16 MB PDF)
