# Supplementary figures and images for: Atomic Analysis of Protein-Protein Interfaces with Known Inhibitors: The 2P2I Database
Source: PLoS One. 2010 Mar 9;5(3):e9598. doi: 10.1371/journal.pone.0009598 (PMC2834754; doi:10.1371/journal.pone.0009598)

**Supplementary Material Figure S1**

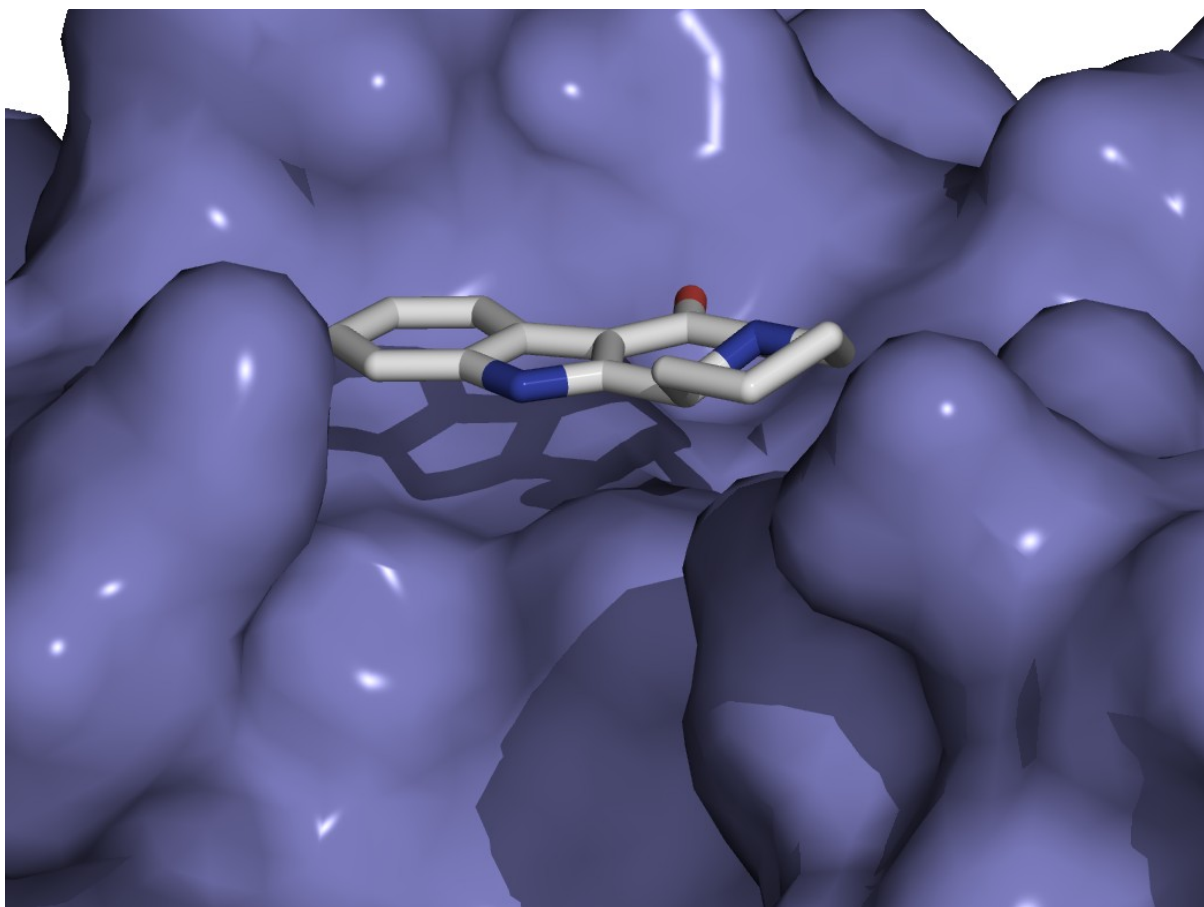

Supplement: Figure S1 — ZipA protein in complex with IQZ inhibitor (PDB code 1S1J). The IQZ inhibitor ZipA surface is shown as a stick representation. (Figure generated with pymol). (0.11 MB PDF) [file pone.0009598.s001.pdf]
