# Supplementary material for: Atomic Analysis of Protein-Protein Interfaces with Known Inhibitors: The 2P2I Database
Source: PLoS One. 2010 Mar 9;5(3):e9598. doi: 10.1371/journal.pone.0009598 (PMC2834754; doi:10.1371/journal.pone.0009598)

Supplementary Material Figure S2

Number of complexes versus Number of Hydrogen bonds/100Å² (±0.1)

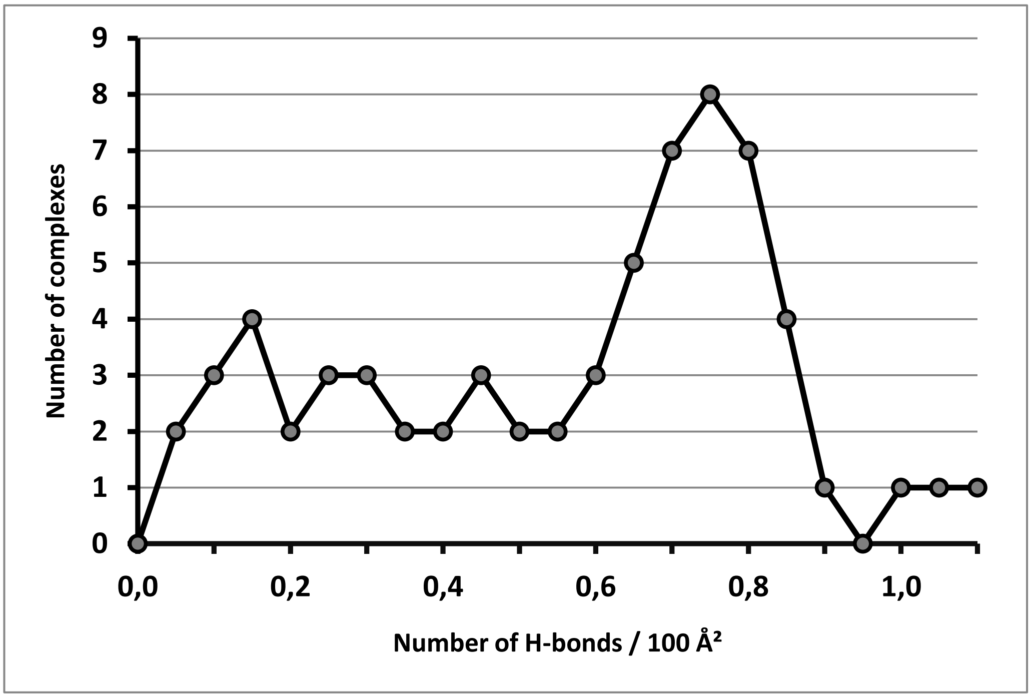

Supplement: Figure S2 — Hydrogen bonds per 100 Å2 of accessible surface area. The X-axis represents the number of hydrogen bonds per 100 Å2. The Y-axis illustrates the number of complexes present in 2P2IDB having this number of hydrogen bonds (within ±0.1), i.e. y value at x = 0.6 indicates that there are 3 complexes having 0.3 to 0.5 hydrogen bonds per 100 Å2. (0.09 MB PDF) [file pone.0009598.s002.pdf]
