# Supplementary material for: Atomic Analysis of Protein-Protein Interfaces with Known Inhibitors: The 2P2I Database
Source: PLoS One. 2010 Mar 9;5(3):e9598. doi: 10.1371/journal.pone.0009598 (PMC2834754; doi:10.1371/journal.pone.0009598)
Supplement: Figure S3 — Advanced query search of the protein databank. This table lists the different parameters used to parse the protein databank to search for proteins bound to a small molecule inhibitor. (0.08 MB PDF) [file pone.0009598.s003.pdf]

Supplementary Material Figure S3

Query used to parse the RCSB PDB

Has Ligand(s)

Has Ligands	Yes

Macromolecular Type

Contains Protein	Yes
Contains DNA	No
Contains RNA	No
Contains	No

Structure Title

Contains	inhibitor

Structure Title

Contains	*

* Protein name (ex: trypsin)
