# Supplementary material for: Atomic Analysis of Protein-Protein Interfaces with Known Inhibitors: The 2P2I Database
Source: PLoS One. 2010 Mar 9;5(3):e9598. doi: 10.1371/journal.pone.0009598 (PMC2834754; doi:10.1371/journal.pone.0009598)
Supplement: Table S1 — Root mean square deviations for the complexes in 2P2I database. The rms are computed between the unbound protein and its equivalent in the protein/protein complex; the unbound protein and its homologous in complex with the inhibitor; the protein in complex with its partner and the homologous in complex with the inhibitor. When several structures are compared, the average is shown. All RMSD were performed over CA atoms with the DaliLite web server. (0.15 MB PDF) [file pone.0009598.s004.pdf]

Supplementary Information Table S1

Root Mean Square Deviations. When sereral structures are compared the average rms is shown.

FREE vs Protein/Protein Complex

Free	ChID	Complex	CHID	rms (CA)
1f46	A	1f47	B	0,90
1z1m	A	1ycq	A	1,80
1z1m	A	1ycr	A	1,90
1r2d	A	1bxl	A	2.3
1f9x	A	1g73	C	2,30
1f9x	A	1nw9	A	2,40
1scn	E	1cse	E	0,30
1scn	E	1r0r	E	0,30
1sup	A	1to2	E	0,30
1hj9	A	2uuy	A	0,70
2zff	H	3b9f	H	0,60
1r6k	A	1tue	B	1,40
2cio	A	3e1z	B	0,40
1m47	A	1z92	A	1,30
na		1oo9	A	
2ppn	A	1b6c	A	0,50
na		2j0t	A	

Class I	Mean	1,86
	Stdev	0,59414
Class II	Mean	0,64
	Stdev	0,42459
ALL	Mean	1,08
	Stdev	0,76478

FREE vs Protein/Inhibitor Complex

Free	ChID	Protein/Inhibitor	CHID	rms (CA)
1f46	A	1s1j,1s1s,1y2f,1y2g	A (1sis:B)	0,70
1z1m	A	1rv1,1t4e	A	2,05
1r2d	A	1ysg,1ysi,1ysn,2o1y,2o2m, 2o2n,2yxj	A	1,79
1f9x	A	2jk7,2opy,3clx,3cm7,3cm2,3eyl	A	2,62
1f9x	A	1tfq,1tft	A	2,40
1scn	E	1bh6	A	0,40
1scn	E	1bh6	A	0,40
1sup	A	1bh6	A	0,50
1hj9	A	2fs8	A	1,60
2zff	H	1jwt	A	0,40
1r6k	A	1r6n	A	0,90
2cio	A	1cvz	A	0,30
1m47	A	1m48	A	0,60
na		1usn	A	
2ppn		1j4r	A	
na		966c	A	

Class I	Mean	1,91
	Stdev	0,7481
Class II	Mean	0,64
	Stdev	0,43074
ALL	Mean	1,13
	Stdev	0,84278

Protein/Protein vs Protein/Inhibitor Complexes

Complex	CHID	Protein/Inhibitor	CHID	rms (CA)
1F47	B	1s1j,1s1s,1y2f,1y2g	A (1s1s:B)	0,85
1YCQ	A	1ttv	A	1,10
1YCR	A	1rv1,1t4e	A	0,95
1BXL	A	1ysg,1ysi,1ysn,2o1y,2o2m, 2o2n,2yxj	A	2,93
1G73	C	2jk7,2opy,3clx,3cm7,3cm2,3eyl	A	0,52
1NW9	A	1tfq,1tft	A	2,10
1cse	E	1bh6	A	0,40
1r0r	E	1bh6	A	0,40
1to2	E	1bh6	A	0,50
2uuy	A	2fs8	A	1,50
3b9f	H	1jwt	A	0,90
1tue	B	1r6n	A	1,50
3e1z	B	1cvz	A	0,40
1z92	A	1m48	A	1,40
1oo9	A	1usn	A	1,20
1b6c	A	1j4r	A	0,61
2j0t	A	966c	A	0,50

Class I	Mean	1,41
	Stdev	0,91668
Class II	Mean	0,85
	Stdev	0,46717
ALL	Mean	1,04
	Stdev	0,6895
