# Supplementary material for: Atomic Analysis of Protein-Protein Interfaces with Known Inhibitors: The 2P2I Database
Source: PLoS One. 2010 Mar 9;5(3):e9598. doi: 10.1371/journal.pone.0009598 (PMC2834754; doi:10.1371/journal.pone.0009598)
Supplement: Table S2 — Secondary structure at interface. This table lists secondary structures at interface (as defined in M&M) for each complex present in 2P2IDB. Information is detailed for both the target protein and its partner. (0.16 MB PDF) [file pone.0009598.s005.pdf]

Supplementary Information Table S2.

Secondary structure at interface, S = sheet, H = helix and C = coil.

		PDB	Target	Partner
CLASS I	ZipA/FtsZ	1f47	S	H
	BclXL/Bak	1bxl	H	H
	MDM2/p53	1ycr	H	H
	MDM2/p53	1ycq	H	H
	XIAP BIR3/SMAC	1g73	S	S
	XIAP BIR3/CASPASE 9	1nw9	S	S

		PDB	Target	Partner
CLASS II	Subtilisin/Eglin C	1cse	C	S
	Subtilisin/Eglin C	1r0r	C	C
	Subtilisin/Eglin C	1to2	C	S
	Trypsin/trypsin inh	2uuy	C	C
	Thrombin/Protein	3b9f	C	C
	HPV E2/HPV E1	1tue	H	H
	Chagasin/papain	3e1z	C	C
	FKBP12/TGFR	1b6c	S	H
	MMP1/TIMP1	2j0t	C	C
	MMP3/TIMP1	1oo9	C	C
	IL-2/IL-2R	1z92	H	S
