# Supplementary material for: Atomic Analysis of Protein-Protein Interfaces with Known Inhibitors: The 2P2I Database
Source: PLoS One. 2010 Mar 9;5(3):e9598. doi: 10.1371/journal.pone.0009598 (PMC2834754; doi:10.1371/journal.pone.0009598)
Supplement: Table S3 — Geometrical and chemical parameters for the subset of the interface at 4.5 Å around the inhibitor. The parameters are detailed for each complex of 2P2IDB and mean and standard deviations are shown for class I, class II and the whole database. (0.16 MB PDF) [file pone.0009598.s006.pdf]

Supplementary Information Table S3

Geometrical parameters for the subset of the interface (4.5 Å around inhibitor)

		planarity	eccentricity	GV_Index	H-bonds	HB/100Å²	Salt-bridges	# Segments	% charged resid
Zip1/FtsZ	1f47*	2,686	0,731	0,76	0	0	0	3	22,22
BclXL/Bak	1bxl*	3,685	0,719	1,65	1	0,12	0	5	23,08
MDM2/p53	1ycr*	2,308	0,745	1	1	0,13	0	2	20,00
MDM2/p53	1ycq*	2,138	0,623	0,54	2	0,30	0	3	0,00
XIAP BIR3/SMAC	1g73*	2,235	0,721	0,71	7	0,99	0	2	42,86
XIAP BIR3/CASPASE 9	1nw9*	2,3	0,631	1,57	5	0,08	0	3	0,00
Class I	mean	2,56	0,70	1,04	2,67	0,39	0	3,00	18,03
	stdev	0,58	0,05	0,47	2,73		0	1,10	16,21
Subtilisin/Eglin C	1cse*	2,02	0,70	1,45	8	0,86	0	6	0,00
Subtilisin/Eglin C	1r0r*	2,13	0,69	1,76	10	1,07	0	6	0,00
Subtilisin/Eglin C	1to2*	2,47	0,70	1,15	10	1,01	0	8	0,00
Trypsin/trypsin inh	2uuy*	2,14	0,81	2,34	9	1,00	1	7	10,00
Thrombin/ProtC_inh	3b9f*	2,70	0,69	1,27	5	0,63	1	7	21,05
HPV E2/HPV E1	1tue*	1,74	0,73	4,42	7	1,17	0	8	27,27
Chagasin/papain	3e1z*	1,97	0,93	1,53	6	0,64	0	8	18,75
FKBP12/TGFR	1b6c*	2,74	0,62	1,83	0	0,00	0	8	21,43
MMP1/TIMP1	2j0t*	2,47	0,35	2,75	10	1,41	0	6	13,33
MMP3/TIMP1	1oo9*	2,35	0,70	1,43	1	0,13	0	7	36,37
IL-2/IL-2R	1z92*	1,37	0,84	1,29	3	0,36	2	6	8,33
Class II	mean	2,19	0,70	1,93	6,27	0,74	0,36	7,00	14,23
	stdev	0,41	0,15	0,96	3,64		0,67	0,89	12,00
ALL	mean	2,32	0,70	1,61	5,00	0,63	0,24	5,59	15,57
	stdev	0,49	0,12	0,91	3,71		0,56	2,18	13,25

Accessible Surface Area for the subset of the interface (4.5 Å around inhibitor)

		INTERFACE ASA	SEPARATED INTERFACES Target	SEPARATED INTERFACES Partner
ZipA/FtsZ	1f47*	1119,3	526,3	593,0
BclXL/Bak	1bxl*	1614,1	766,4	847,7
MDM2/p53	1ycr*	1173,9	529,8	644,1
MDM2/p53	1ycq*	1020,2	461,3	558,9
XIAP BIR3/SMAC	1g73*	679,8	288,9	390,9
XIAP BIR3/CASPASE 9	1nw9*	754,8	313,8	441,1
	Class I	1060,3	481,1	579,3
		335,5	173,9	162,1
Subtilisin/Eglin C	1cse*	1064,3	474,1	590,2
Subtilisin/Eglin C	1r0r*	1040,3	466,9	573,4
Subtilisin/Eglin C	1to2*	1130,5	514,9	615,5
Trypsin/trypsin inh	2uuy*	969,6	415,8	553,8
Thrombin/ProtC_inh	3b9f*	1103,8	503,3	600,5
HPV E2/HPV E1	1tue*	595,8	289,6	306,1
Chagasin/papain	3e1z*	1129,1	606,5	522,7
FKBP12/TGFR	1b6c*	1286,6	631,7	654,9
MMP1/TIMP1	2j0t*	1040,4	473,3	567,1
MMP3/TIMP1	1oo9*	1121,2	567,5	553,7
IL-2/IL-2R	1z92*	1344,5	676,7	667,7
	Class II	1075,1	510,9	564,1
		192,3	108,3	96,0
	ALL	1069,9	500,4	569,5
		241,5	130,3	118,5
